# Stabilization of soil organic matter in Luvisols under the influence of various tree species in temperate forests

**DOI:** 10.1038/s41598-025-85883-6

**Published:** 2025-01-08

**Authors:** Karolina Staszel-Szlachta, Ewa Błońska, Jarosław Lasota

**Affiliations:** Department of Ecology and Silviculture, Faculty of Forestry, University of Agriculture in Krakow, 29 Listopada 46 Str, Krakow, 31-425 Poland

**Keywords:** Base cations, Forest ecosystem, Luvisols, Organic matter fractions, Biogeochemistry, Environmental sciences

## Abstract

Tree species through aboveground biomass and roots are a key factors influencing the quality and quantity of soil organic matter. Our study aimed to determine the stability of soil organic matter in Luvisols under the influence of five different tree species. The study areas were located 25 km north of Krakow, in southern Poland. The study included five tree species - Scots pine (*Pinus sylvestris* L.), European larch (*Larix decidua* Mill.), pedunculate oak (*Quercus robur* L.), beech (*Fagus sylvatica* L.) and hornbeam (*Carpinus betulus* L.). Forest stands growing in the same soil conditions (Luvisols) with similar geological material (loess) and grain size were selected for the study. We evaluated labile and heavy fractions of soil organic matter (SOM). Additionally, basic physicochemical properties (pH, carbon and nitrogen content, base cation content) were determined in soil samples. The results of our study showed that soils under the influence of coniferous species were characterized by a higher content of carbon of free light fraction (C_fLF_) and carbon of occluded light fraction (C_oLF_) compared to deciduous species. Similar relationships were found with the nitrogen content of the free light fraction (N_fLF_) and nitrogen of occluded light fraction (N_oLF_). Higher C_MAF_ and N_MAF_ contents were recorded in soils influenced by deciduous species. The carbon, nitrogen and base cations content positively correlated with the C and N of free light fraction and occluded light fraction. PCA analysis confirmed the connection of C and N of heavy fractions (C_MAF_ and N_MAF_) with deciduous species. Our research shows that avoiding single-species conifer stands and introducing admixtures of deciduous species, which increase SOM, is justified in forest management. The selection of suitable species will provide greater stand stability and contribute more to the carbon accumulation in the soil.

## Introduction

Soils play a key role as carbon reservoirs, which has a significant impact on the regulation of the carbon cycle. Carbon stored in soils is mainly in the form of dead plant and animal matter, and microorganisms are involved in its decomposition. The soil’s ability to sequester carbon depends on a number of factors, such as soil type, moisture content, temperature, and management^1^. Additionally, grain size plays an important role in carbon sequestration, as the ability to form complexes with organic matter is directly proportional to the content of fine fractions in the soil^2,3^. Organic matter bound to silt and clay is more stable, which limits its availability to microorganisms and enzymes, thus slowing its mineralization^4^. Soil pH affects the activity of microorganisms and the dynamics of element cycling, which directly affects the rate of organic matter decomposition^5,6^. In turn, minerals such as iron (Fe) and aluminum (Al) can bind organic matter to form stable mineral-organic complexes. These complexes are less prone to decomposition, which promotes long-term carbon sequestration^7^. The chemical and physical properties of soils are influenced by the quantity and quality of litter provided, the ability of roots to take up nutrients, and the microclimate inside the forest^8^. Therefore, forest management and the selection of appropriate tree species are crucial in raising the soil’s potential for greater stabilization of organic C in the soil mineral layer^9^.

Decomposition of organic matter is one of the most important processes that affect element cycling and carbon storage^10^. Since fine roots make up the bulk of underground biomass, their decomposition has a significant impact on soil nutrient availability through faster turnover and higher metabolic activity^11^. The decomposition rate of deciduous species is faster compared to coniferous species due to their lower lignin and calcium content, as well as other secondary metabolites that affect microbial decomposition^12^. Soil organic matter (SOM) consists of the labile fraction (fLF – free light fraction), which is not bound to soil mineral particles and consists mainly of undecomposed or partially decomposed plant debris, fine roots, and mycelium^7^. By not being bound to soil minerals, it is readily available to microorganisms^13^. Other fraction types include the occluded light fraction (oLF) and the mineral associated fraction (MAF). The light occluded fraction, although similar to the free light fraction in density, is more stable because soil aggregates protect it from rapid decomposition^14^. As a result, the mineralization process of this fraction is slower, allowing it to retain carbon in the soil longer^15,16^. The MAF fraction of soil organic matter is resistant to decomposition and microbial transformation^17^. The MAF fraction plays a fundamental role in the long-term stabilization of soil carbon. Due to the strong chemical bonds between organic matter and soil minerals, this fraction contributes to the permanent retention of carbon in the soil environment^18^. Carbon bound in unstable organic matter fractions is short-term, subject to frequent changes in the soil environment, while in heavy fractions it can remain in the soil for decades^19^.

In the era of climate change, there is an increased frequency of weather extremes, and seasonal anomalies can negatively impact the soil environment and forest ecosystems, affecting their stability^20^. It is important to know the influence of tree species on the fractional composition of soil organic matter. Research on carbon sequestration in forest soils is crucial for sustainable management of forest resources and combating climate change. The purpose of our study was to determine the effects of five different tree species on the fractional composition of soil organic matter in Luvisols. Additionally, attempts were made to determine the relationship between the fractional composition of soil organic matter and the activity of enzymes involved in the cycling of C, N and P. The study included primary tree species: Scots pine (*Pinus sylvestris* L.), European larch (*Larix decidua* Mill.), Pedunculate oak (*Quercus robur* L.), European beech (*Fagus sylvatica* L.) and European hornbeam (*Carpinus betulus* L.) occurring in stands of temperate climates^21^. The fractional composition of soil organic matter was correlated with basic soil properties and enzymatic activity. In the study, it was assumed that deciduous and coniferous species significantly differ in the fractional composition of soil organic matter. Furthermore, it was hypothesized that the physico-chemical characteristics of soil are closely associated with the fractional composition of soil organic matter. The study also posited that the diversity in the fractional composition of soil organic matter, influenced by the type of tree species, directly impacts soil enzymatic activity. We hypothesize that the various fractions of organic matter exhibit differing degrees of availability to microorganisms, which are the primary agents responsible for enzyme production. We assume that the type of organic matter fraction plays a pivotal role in shaping microbial dynamics and, consequently, the enzymatic processes within the soil.

## Materials and methods

### Study sites

**Fig. 1:**
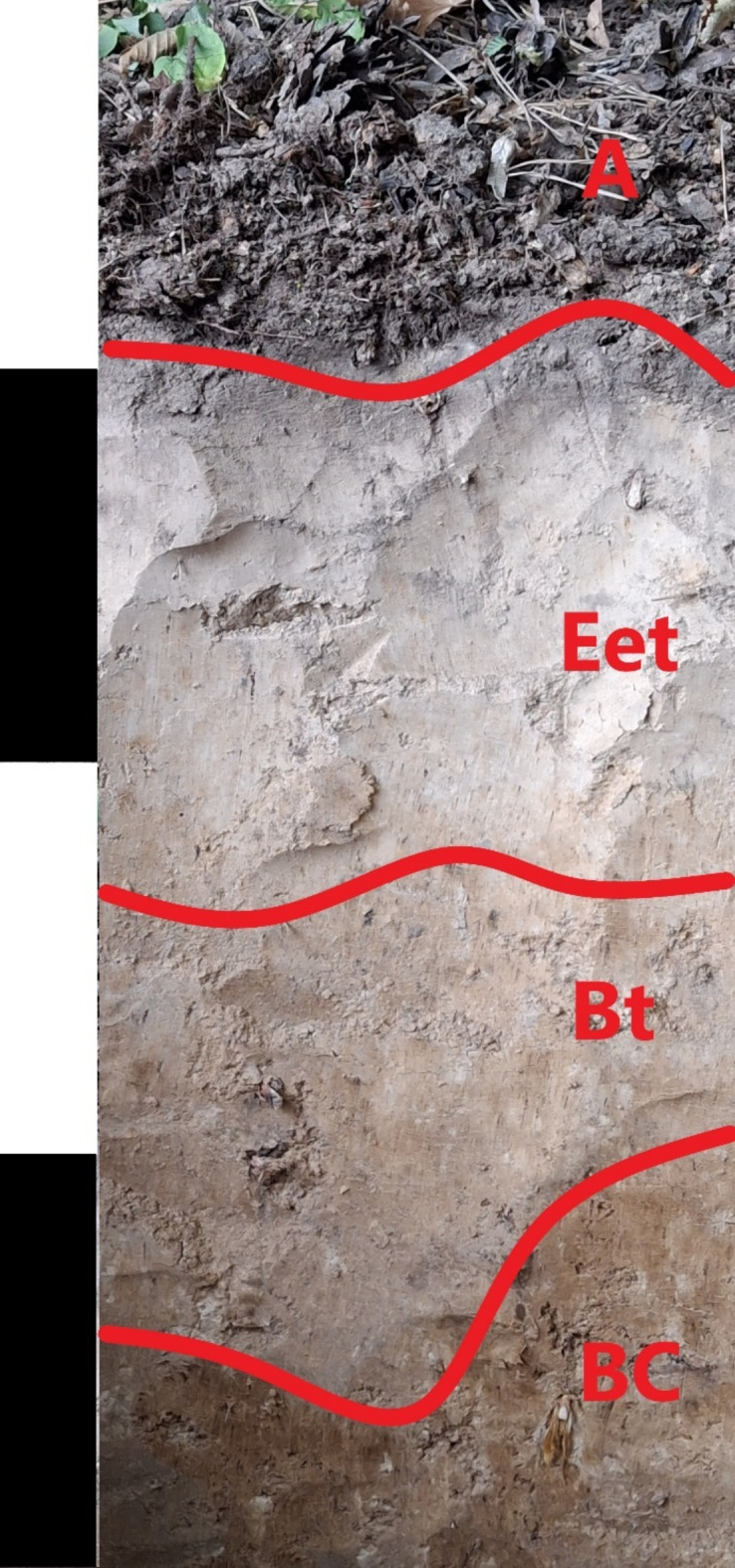
Soil profile removed from the study area.

The study was carried out on experimental plots belonging to the Department of Ecology and Silviculture at the University of Agriculture in Kraków, Miechów Forest District, Poland (50º11.46.35 N, 20º3.54.28E). The study covered five species of trees––Scots pine (*Pinus sylvestris L.)*, European larch *(Larix decidua Mill.)*, English oak (*Quercus robur L.)*, European beech (*Fagus sylvatica L.)* and European hornbeam *(Carpinus betulus L.*). The research areas were located in one large forest complex with an area of ​​approximately 1000 ha. Within it, single-species stands of Scots pine, European larch, English oak, European beech and European hornbeam were distinguished. The forest stands contained single species, without admixtures of other species, of similar age (70–80 years) and stand density. Before designating the research areas, we studied existing documentation of the area, especially geological maps. Analysis of the materials confirmed that the research area includes loess formations. The study area was characterized by the same geological material (loess) and texture, in addition the area is subject to the same silvicultural treatments appropriate to the developmental phase of the stand. The whole area was characterized by the presence of Luvisols (IUSS-WRB soil classification^22^ developed from homogeneous loess) (Fig. 1). The soil profile described in the study area has the following sequence of horizons: A-Eet-Bt-BC. In the deeper horizon, i.e. BC, the occurrence of calcium was noted. The studied area was characterized by similar contents of sand, silt and clay (average contents were 16%, 76%, 8%, respectively). Study plots were selected in a 1000 ha forest complex. Each of the five plot variants (0.1 ha study plots) was studied in twenty-five replicates. A total of 125 study plots (five tree species × twenty-five repetitions = 125 study plots) were used for the study. In each test points, soil samples were taken for soil properties analysis, and in addition, soil samples were taken for enzymatic activity analysis. Soil samples were taken from the surface horizon from the A horizon, which were humus-mineral horizon 15 cm thick after removal of the organic horizon. Soil samples for laboratory analyses were taken at a location approximately 100 cm from the stumps of the trees of the species under study, within the range of their root systems. Sampling and analysis was performed in 2022.

### Chemical analysis

Soil samples were dried at 65 °C for 72 h, then all samples were sieved through a 2 mm sieve. Physicochemical properties were determined in the prepared samples: pH was determined by potentiometric method in water. Total nitrogen and carbon content was determined using LECO CNS True Mac Analyser (Leco, St. Joseph, MI, USA). ICP-OES (iCAP 6500 DUO, Thermo Fisher Scientific, Cambridge, Great Britain) was used to determine the content of base cations (Ca2+, Mg2+, K+, Na+). Fresh soil samples with natural moisture were sieved through a 2 mm sieve and stored at + 4 °C to determine enzyme activity. The activities of extracellular enzymes β-glucosidase (BG), β-D-cellobiosidase (CB), β-xylosidase (XYL), N-acetyl-β-D-glucosaminidase (NAG), phosphatase (PH) and arylsulfatase (SP) were determined using fluorogenically labeled substrates. Fluorescence was measured on a plate reader at an excitation wavelength of 355 nm and an emission wavelength of 460 nm. Dehydrogenase (DH) activity was determined using the Lenhard method according to the Casidy procedure^23^. Physical fractionation of SOM was performed according to the method described by Sohi et al.^24^. A 10-g soil sample was placed in a 200-ml centrifuge tube and 90 ml of NaI (1.7 g cm-3) was added. Each sample was sonicated (60 watts for 200 s) to destroy aggregates, shaken for 1 min, and centrifuged for 30 min. The free light fraction (fLF) was removed by pipetting and collected on a glass fiber filter. The soil remaining at the bottom of the centrifuge tubes was mixed with another 90 ml of NaI. After centrifugation, the material released from the aggregate-occluded light fraction (oLF) was collected on a glass fiber filter. The remaining fraction was assumed to consist of the mineral associated fraction (MAF) of SOM^25^. The fractions were analyzed for carbon and nitrogen (CfLF, CoLF, CMAF, NfLF, NoLF, and NMAF, respectively) using a LECO CNS True Mac Analyzer.

### Statistical analysis

One-way analysis of variance was used to find the significance of differences between the mean values of the analyzed chemical parameters, soil fractional composition and enzymatic activity according to the stands. The normality of the distribution was checked using the Shapiro-Wilk test. A post-hoc test was used to evaluate differences between the mean values of the characteristics. Results were considered statistically significant at < 0.05. Spearman correlation coefficients were calculated between individual soil organic matter fractions and soil properties and enzymatic activity. Principal component analysis (PCA) was used to group variables by tree species, which included chemical properties, soil biological properties and individual soil organic matter fractions. Using PCA analysis, we identified the variables that most contribute to the variability of the data. All statistical analyses were performed using R statistical software (R Core Team, 2022), R Studio^26^ and Statistica 13 software^27^.

## Results

**Fig. 2:**
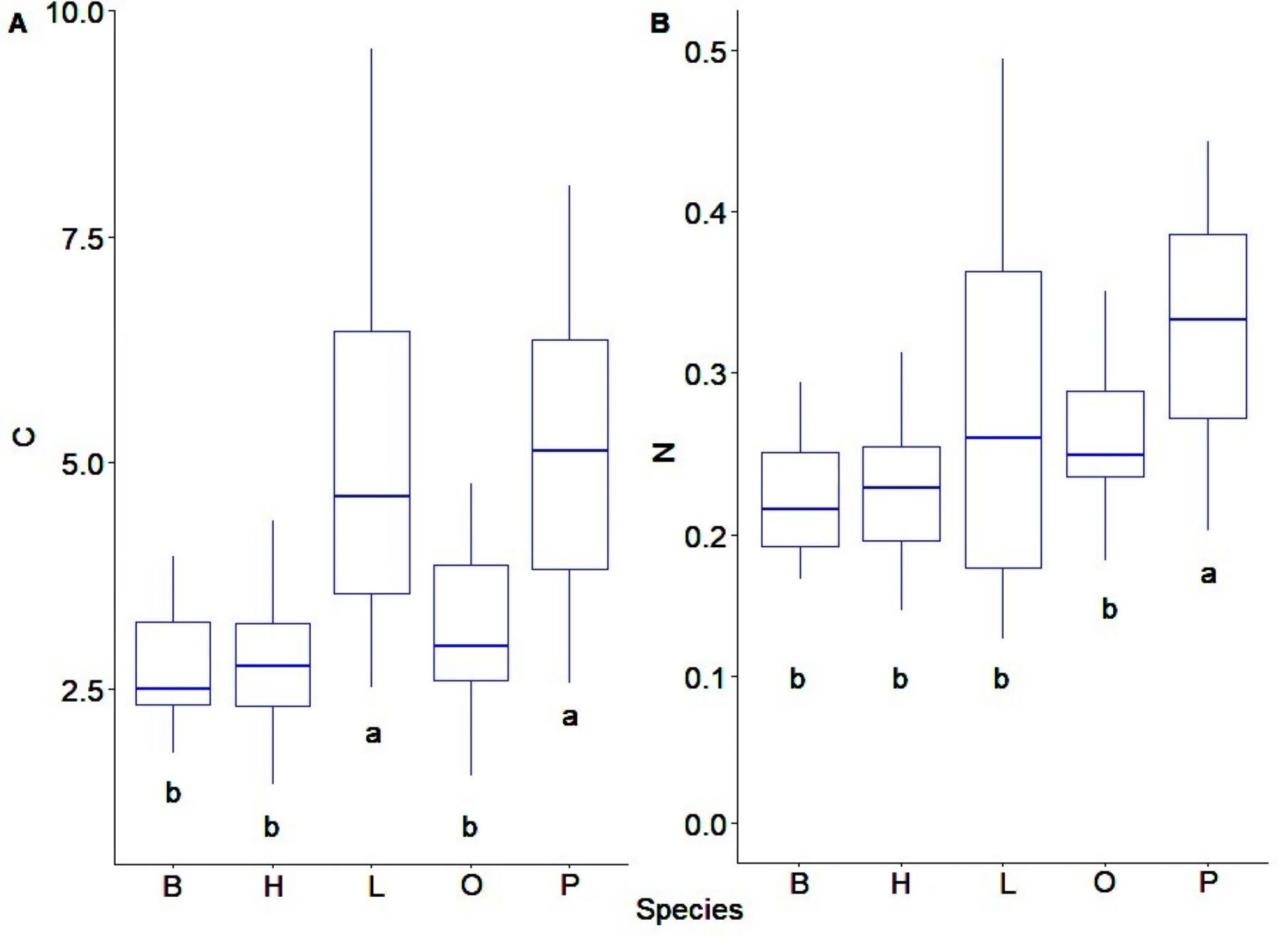
Content of carbon - C and nitrogen - N (%) in soils of various forest stands; lowercase letters (**a**, **b**) indicate significant differences between the species covered by the study B-beech, H-hornbeam, L-larch, O-oak, P-pine.

The highest average C content was recorded in the soils of pine and larch stands (5.17 and 4.96%, respectively) and it differed significantly from the C content in the soils of oak, hornbeam and beech stands (3.30, 2.80 and 2.70%, respectively) (Fig. 2a, b). For N content, the soils of pine stands showed significant differences from the soils influenced by the other stands (Fig. 2). The highest pH was recorded in the soils of beech and hornbeam stands (4.25 and 4.28, respectively), while the lowest pH was recorded in the soils of larch stands (3.81). Ca content ranged from 2.13 cmol(+)·kg^− 1^ in the soils of pine stands to 1.18 cmol(+)·kg^− 1^ in the soils of hornbeam stands. The soils of larch stands differ from those of other stands and showed the lowest Mg content. In addition, the soils of conifer stands differed from those of deciduous stands in terms of Na content. Soils of coniferous stands showed the highest BC values, and soils of hornbeam stands showed the lowest (Table 1). Analysis of the content of individual micronutrients in the studied soils showed statistically significant differences between the studied tree species. The highest contents of Al, Cu, Fe, P, Pb and Zn were recorded in soils of coniferous stands, while the highest contents of Cd, Co, Mn were recorded in soils of beech and hornbeam stands (Table 2).

**Table 1:** Table 1 Basic physicochemical properties of the tested soils depending on the species.

	pH_H20_	C	*N*	Ca	K	Mg	Na	BC
Larch	3.81 ± 0.13^c^	4.96 ± 1.95^a^	0.27 ± 0.11^b^	1.37 ± 0.59^b^	0.11 ± 0.04^c^	0.26 ± 0.10^c^	0.03 ± 0.01^a^	1.78 ± 0.71^b^
Beech	4.25 ± 0.15^a^	1.70 ± 0.70^b^	0.14 ± 0.05^b^	1.26 ± 0.65^b^	0.14 ± 0.05^bc^	0.27 ± 0.12^bc^	0.02 ± 0.00^b^	1.69 ± 0.82^b^
Hornbeam	4.28 ± 0.18^a^	2.80 ± 0.68^b^	0.22 ± 0.04^b^	1.18 ± 0.59^b^	0.14 ± 0.05^bc^	0.34 ± 0.15^bc^	0.02 ± 0.00^b^	1.67 ± 0.77^b^
Pine	3.98 ± 0.18^b^	5.17 ± 1.57^a^	0.33 ± 0.07^a^	2.13 ± 0.78^a^	0.19 ± 0.07^a^	0.47 ± 0.12^a^	0.03 ± 0.01^a^	2.82 ± 0.94^a^
Oak	3.94 ± 0.20^b^	3.30 ± 1.03^b^	0.26 ± 0.06^b^	1.29 ± 0.66^b^	0.16 ± 0.06^ab^	0.37 ± 0.12^ab^	0.02 ± 0.01^b^	1.84 ± 0.82^b^

mean ± SD; C and N (%); Ca, K, Mg, Na, BC-sum of cations (cmol(+)·kg^− 1^); (a, b) mean significant differences between tree species.

**Table 2:** The content of microelements (mg/kg) in the tested soils depending on the species analyzed.

	Al.	Cd	Co	Cr	Cu	Fe	Mn	Ni	*P*	Pb	Zn
Larch	13657.20± 907.32^a^	0.30± 0.12^ab^	3.67± 0.50^cd^	25.29± 2.35^a^	8.88± 3.05^a^	9142.75± 1212.39^ab^	150.82± 34.25^c^	6.90± 2.00^c^	315.83± 109.25^a^	61.35± 24.62^ab^	44.33± 12.59^a^
Beech	13188.40± 1262.87^ab^	0.37± 0.13^a^	4.29± 0.74^a^	2.61± 0.43^c^	6.19± 1.44^b^	8761.12± 933.25^ab^	486.82± 247.46^a^	9.27± 1.30^ab^	287.65± 63.80^ab^	54.21± 16.14^bc^	41.04± 10.04^ab^
Hornbeam	12572.40± 1099.26^bc^	0.38± 0.14^a^	4.14± 0.74^ab^	25.17± 2.09^a^	5.93± 0.81^b^	8527.88± 995.48^bc^	364.07± 190.58^ab^	9.30± 1.97^ab^	267.69± 51.49^ab^	41.85± 13.74^c^	35.64± 9.55^b^
Pine	13689.00± 1088.58^a^	0.34± 0.09^a^	3.79± 0.43^bc^	14.39± 14.64^b^	8.32± 1.67^a^	9591.78± 1384.85^a^	273.54± 101.96^bc^	10.20± 1.89^a^	306.54± 67.20^a^	68.36± 17.10^a^	46.34± 9.63^a^
Oak	12069.16± 1520.95^c^	0.22± 0.06^b^	3.26± 0.50^d^	25.59± 3.34^a^	5.95± 0.96^b^	7854.38± 1188.54^c^	286.06± 147.13^b^	8.25± 1.43^bc^	244.68± 56.94^b^	48.97± 13.23^bc^	34.67± 6.92^b^

mean ± SD; (a, b) mean significant differences between tree species.

The highest CB activity was recorded in soils of oak and hornbeam stands (3.91 and 3.20 nmol MUB g^− 1^·C·h^− 1^, respectively) and the lowest in soils of beech stands (0.54 nmol MUB g^− 1^·C·h^− 1^). Soils of larch stands differed from those of other stands in terms of BG and NAG activity. The soils under larch showed the highest values of 34.03 and 26.23 nmol MUB g^− 1^·C·h^− 1^. For XYL activity, the values ranged from 8.67 to 0.89 nmol MUB g^− 1^·C·h^− 1^ in the soils of pine and beech stands, respectively. The highest PH and SP activities were recorded in soils of beech stands and the lowest in soils of larch stands (Table 3).

**Table 3:** Enzymatic activity (nmol MUB g^− 1^ ·C ·h^− 1^) of soils in various forest stands.

	CB	BG	NAG	XYL	PH	SP
Larch	2.12 ± 1.70^ab^	34.03 ± 16.78^a^	26.23 ± 13.24^a^	7.75 ± 4.69^a^	46.18 ± 18.85^c^	0.99 ± 1.98^b^
Beech	0.54 ± 0.74^b^	12.90 ± 10.32^b^	11.77 ± 5.36^cd^	0.89 ± 0.92^b^	100.36 ± 20.52^a^	2.27 ± 2.35^a^
Hornbeam	3.20 ± 2.46^a^	11.12 ± 4.97^b^	10.45 ± 3.65^d^	1.66 ± 1.53^b^	80.38 ± 29.37^ab^	1.32 ± 1.18^ab^
Pine	2.48 ± 2.36^ab^	13.29 ± 8.59^b^	21.39 ± 11.48^ab^	8.67 ± 4.54^a^	87.02 ± 48.73^a^	1.28 ± 1.01^ab^
Oak	3.91 ± 4.35^a^	14.99 ± 13.61^b^	17.69 ± 8.53^bc^	7.23 ± 3.36^a^	58.77 ± 24.88^bc^	1.15 ± 1.08^ab^

mean ± SD; CB—β-D-cellobiosidase,, BG—β-glucosidase, NAG—N-acetyl-β-D-glucosaminidase, XYL—β-xylosidase, PH—phosphatase, SP – arylsulphatase; (a, b) mean significant differences between tree species.

**Fig. 3:**
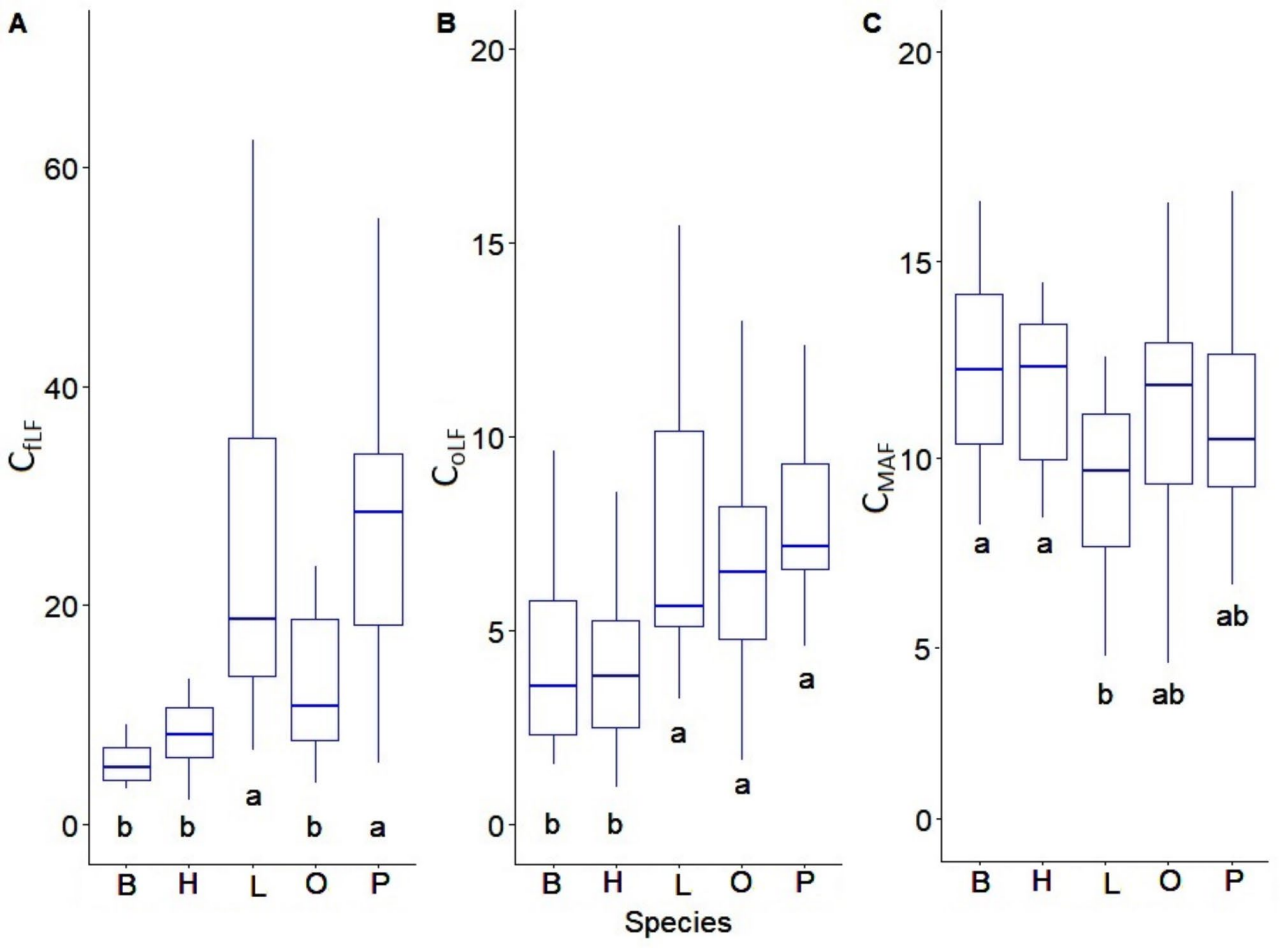
Carbon content in soil organic matter fractions in soils of various forest (C_fLF_ - carbon of free light fraction (g.kg^− 1^), C_oLF_ – carbon of occluded light fraction (g.kg^− 1^), C_MAF_ – carbon of mineral associated fraction (g.kg^− 1^); B-beech, H-hornbeam, L-larch, O-oak, P-pine; (**a**, **b**) mean significant differences between tree species.

**Fig. 4:**
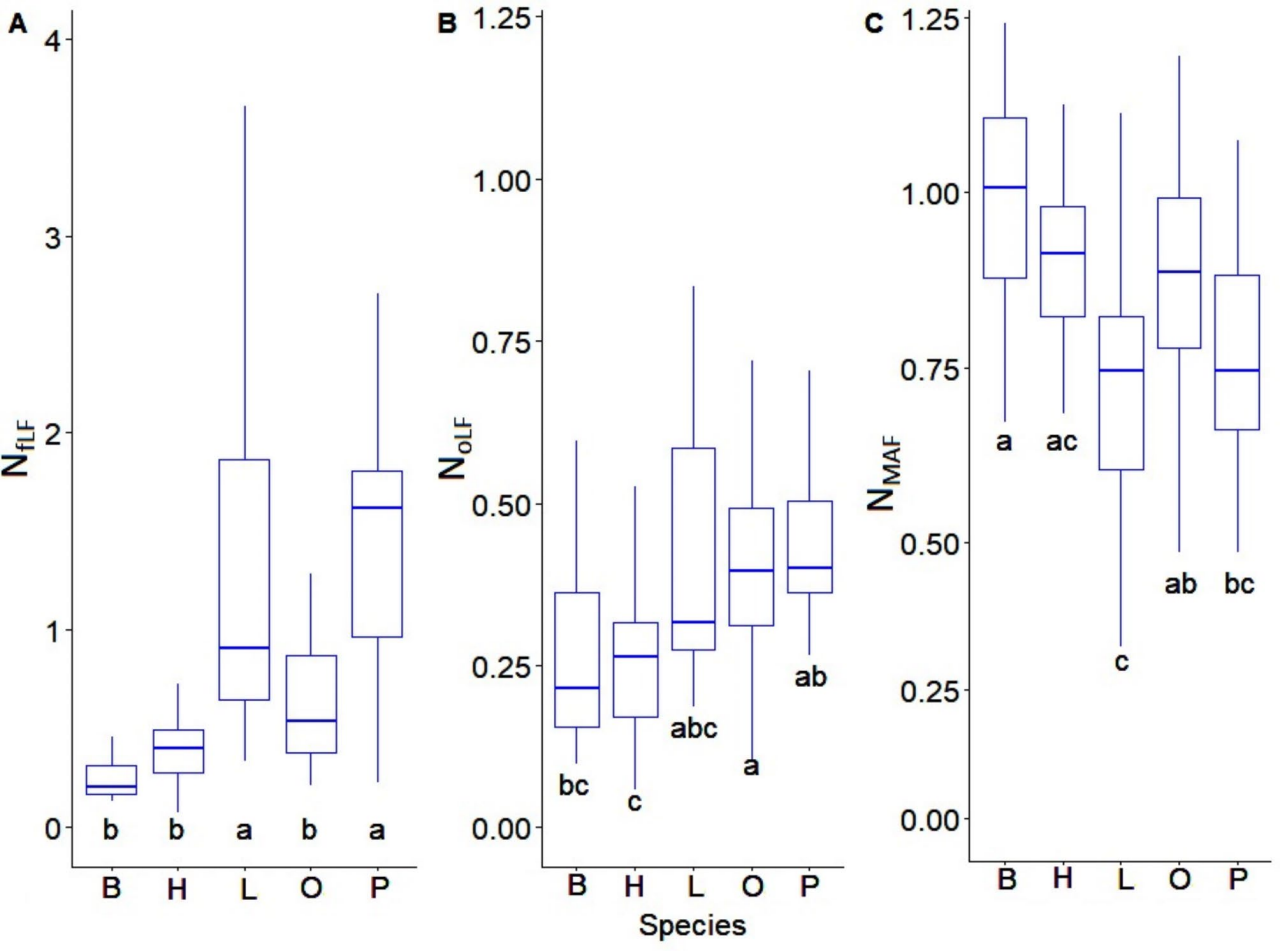
Nitrogen content of soil organic matter fractions in soils of different stands (N_fLF_ - nitrogen of free light fraction (g.kg^− 1^), N_oLF_ – nitrogen of occluded light fraction (g.kg^− 1^), N_MAF_ – nitrogen of mineral associated fraction (g.kg^− 1^); B-beech, H-hornbeam, L-larch, O-oak, P-pine; (**a**, **b**, **c**) mean significant differences between tree species.

**Fig. 5:**
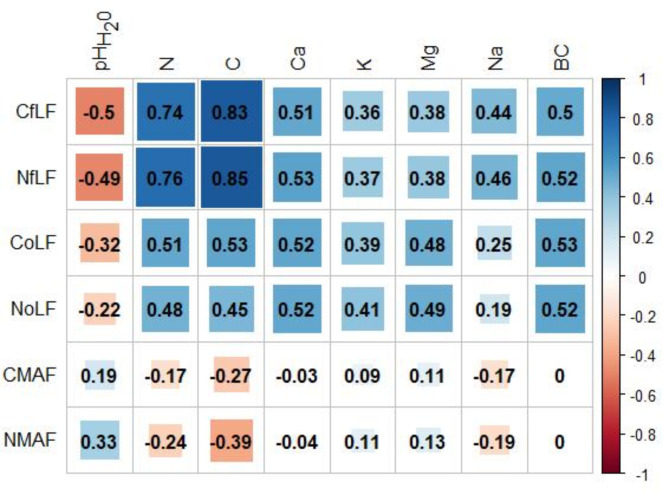
Relationships between the fractional composition of soil organic matter and the basic physicochemical properties of the tested soils (significant correlations above 0.32).

**Fig. 6:**
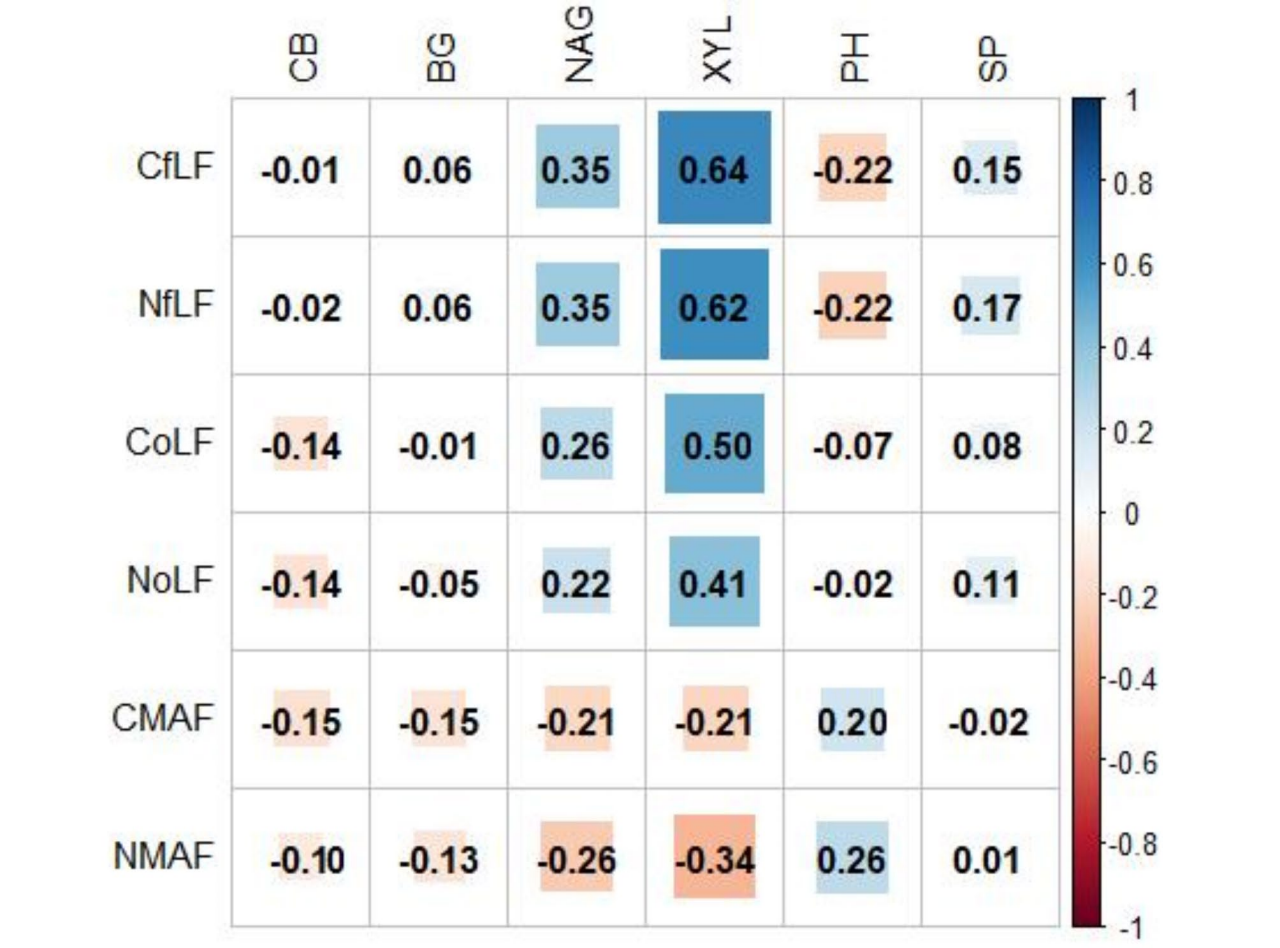
The relationship between the fractional composition of soil organic matter and the enzymatic activity of the tested soils (significant correlations above 0.32).

**Fig. 7:**
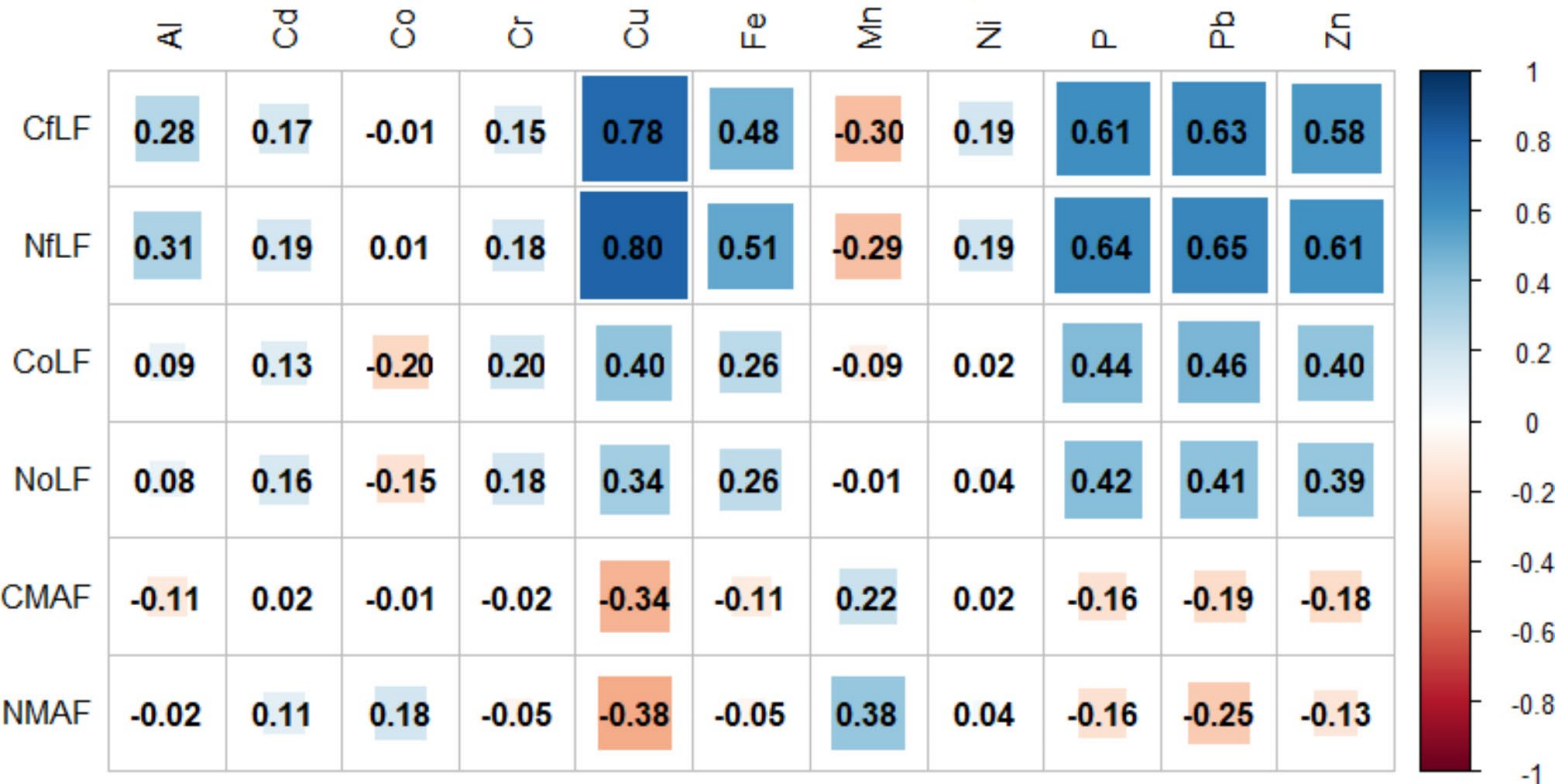
Correlation between the fractional composition of soil organic matter and the content of microelements in the soil (significant correlations above 0.32).

**Fig. 8:**
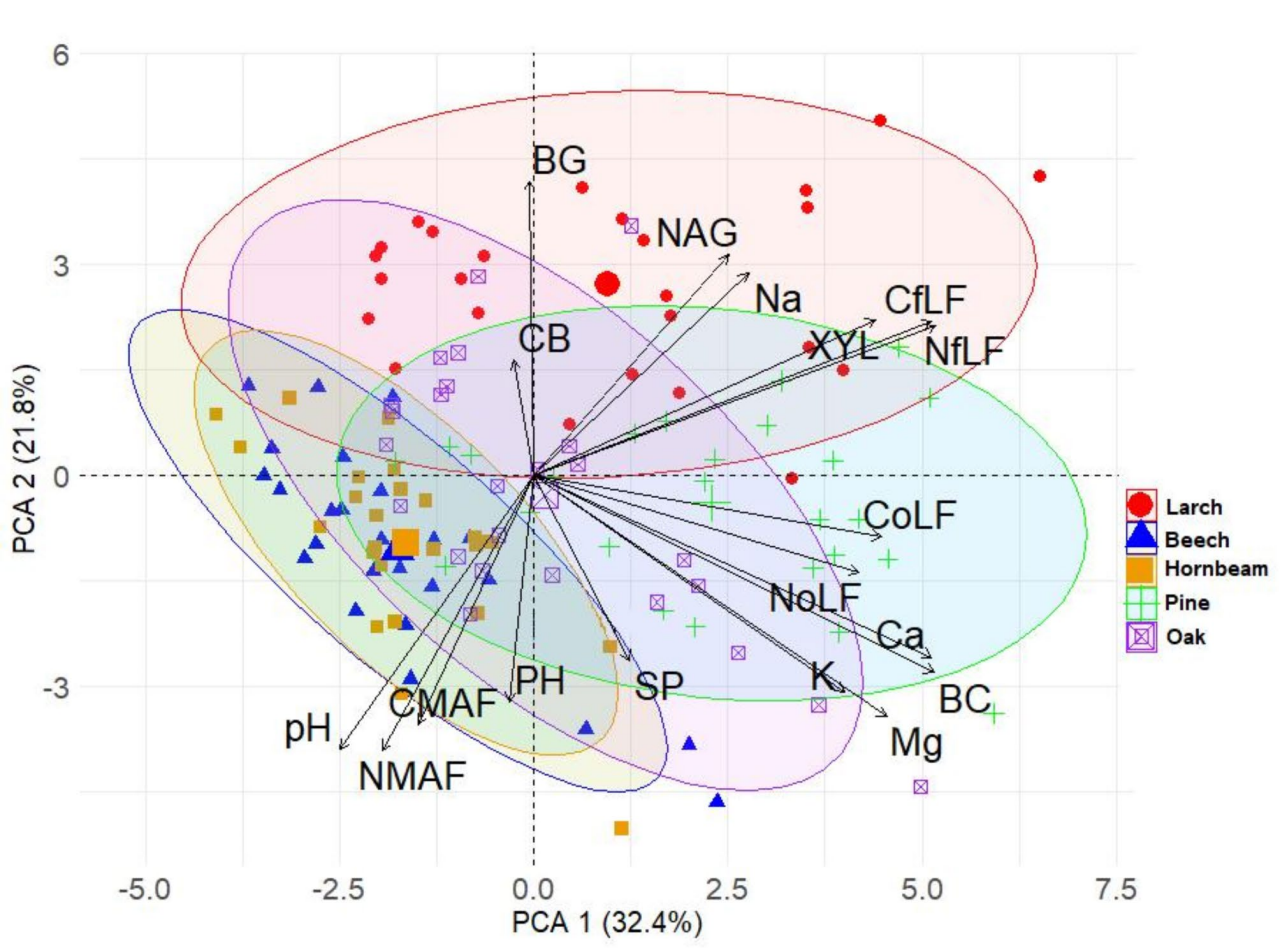
Projection of variables onto the plane of the first and second PCA factors. (pH, Ca, K, Mg, Na BC-sum of cations (cmol(+)·kg^− 1^); CB—β-D-cellobiosidase,, BG—β-glucosidase, NAG—N-acetyl-β-D-glucosaminidase, XYL—β-xylosidase, PH—phosphatase, SP – arylsulphatase; C_fLF_ - carbon of free light fraction (g.kg^− 1^), C_oLF_ – carbon of occluded light fraction (g.kg^− 1^), C_MAF_ – carbon of mineral associated fraction (g.kg^− 1^), stands (N_fLF_ - nitrogen of free light fraction (g.kg^− 1^), N_oLF_ – nitrogen of occluded light fraction (g.kg^− 1^), N_MAF_ – nitrogen of mineral associated fraction (g.kg^− 1^)).

The highest C_fLF_ contents were found in soils with coniferous species and they show statistically significant differences with respect to soils with deciduous species. The lowest contents were found in soils with beech and hornbeam (Fig. 3A). Soils with pine, larch and oak differ significantly from soils with hornbeam and beech in terms of C_oLF_ content, (Fig. 3B). The heavy fraction of C_MAF_ in soils with beech and hornbeam stands differs significantly from soils with larch stands (Fig. 3C). The amount of N_fLF_ differed significantly between soils with coniferous and deciduous species. The highest N_fLF_ values were recorded in soils of larch stands, and the lowest in soils of beech stands (Fig. 4A). The N_oLF_ content showed differences among species. The highest values were recorded in soils of larch stands (Fig. 4B). In contrast, higher N_MAF_ content was recorded in soils of deciduous species compared to soils of coniferous species (Fig. 4C). The correlations between the fractional composition of soil organic matter and the basic physicochemical properties of the studied soils were strong. There was a positive strong correlation between the light fraction (fLF) and the occluded light fraction (oLF) and C and N content. A negative correlation with soil pH was also found for these fractions (Fig. 5). NAG and XYL activity showed a positive correlation with the light fraction (fLF) and the occluded light fraction (oLF), C and N (Fig. 6). The content of Cu, Fe, P, Pb, Zn in the soil positively correlated with the light fraction (fLF) and the occluded light fraction (oLF). The heavy fraction (MAF) negatively correlates with Cu content while positively correlates with Mn content (Fig. 7). The principal component analysis PCA conducted explains in total 47.58% of the variation. The analysis confirmed the distinctiveness of deciduous and coniferous stands (Fig. 8). Soils of pine and larch stands are associated with a higher content of free and occluded light fraction, while soils of deciduous stands are associated with a higher content of heavy fraction. Soils of deciduous stands were characterized by significantly higher pH. PCA analysis confirmed higher pH in the soils of beech and hornbeam stands, and at the same time the soils of these stands were characterized by high PH and SP activity (Fig. 8).

## Discussion

### Influence of species on the chemical properties of soils

The experiment conducted indicates the significant influence of stand species composition in shaping the fractional composition of SOM. The analyzed stands grew under similar climatic and soil conditions, thanks to which it was possible to capture the influence of individual species on detailed soil properties. Soils of deciduous such as beech, oak or hornbeam and coniferous species differ significantly in the fractional composition of SOM. Deciduous and coniferous tree species provide different types of organic residues to the soil, which have different chemical structures and decomposition rates which consequently affect the quantity and quality of SOM^28,29^. Soils under deciduous trees such as beech and hornbeam were characterized by higher pH and higher nutrient availability, which promoted faster decomposition of organic matter, formation of the more stable SOM fraction stronger bound to mineral fractions and faster mineralization. In pine and larch stands, the light fraction of SOM was dominant because needles and other organic residues contain more lignin and phenolic compounds, which slows their decomposition^30^. As a result, the light fraction under conifers is less dynamic and limitation of mineralization, and there is a lower proportion of fractions that form more permanent connections with the mineral fraction of the soil. In our study, we found higher C content in coniferous stands due to slower decomposition rates due to high lignin content^31,32^. Coniferous stands are generally less abundant in macronutrients such as N, P, K, Ca and Mg than deciduous stands. The study by Laganiere et al.^33^ demonstrated an increase of up to 25% in humus-associated C stocks by introducing deciduous species, this is because rapidly decomposing leaf litter is immediately stabilized in mineral horizons^34^. In addition, the content of Fe and Al oxides and hydroxides affect the adsorption of organic compounds into the soil slowing down their decomposition. In our study, we recorded higher Fe and Al. values in coniferous stands which may have a direct bearing on the sorption complex (BC). Similar relationships have been reported in other studies where the sorption of organic compounds to clay minerals and Fe and Al oxides and hydroxides was found to be strongly pH dependent^35,36^.

### Effects of individual tree species on soil organic matter fractions and microbial community

Soils of beech stands are characterized by a higher proportion of the heavy SOM fraction compared to other species, especially conifers. Similar relationships were observed in the soils of mountainous areas, where the variation in the proportion of individual fractions was analyzed depending on the tree species and altitude location. In this experiment, the content of the fraction bound to mineral particles (MAF) was found to be higher, up to 50%, in beech stands at the same position in the altitude gradient as fir stands^37^. In the study area, the occurrence of calcium in deeper horizons was noted, which is taken up by beech trees and transported to the surface horizons. In addition, Ca rich beech litter contributes to a healthy soil structure by helping to loosen and stabilize organic matter, which increases the soil’s ability to retain water and essential nutrients^38^. The turnover of basic nutrients in the soil depends on the availability of elements, the buffering capacity of the soil, and the activity of the rhizosphere of different tree species^39^. The higher accumulation of C in mineral horizons may be due to a higher concentration of fine roots and, at the same time, increased enzymatic activity of soil microorganisms stimulated by the diversity of litter^40^. The study by Staszel-Szlachta et al.^10^ demonstrated differences in the composition of bacterial and fungal communities in the soil of deciduous species compared to coniferous species such as pine and larch. Soil samples obtained in ash stands differed in the number and diversity of microorganisms compared to soils under the influence of other species. The increase in microbial activity is influenced by dead organic matter from the roots. Then, decomposing fine roots affect mineralization and root decomposition processes in the soil profile. In addition, the amount of nutrients released as a result of the death of this fraction of roots is much higher than as a result of the decomposition of plant residues^37,41^. In addition, root secretions, which are used by microorganisms as a carbon source, are considered a key determinant of microbial structure in the rhizosphere^42^. As a result, the secretions play an important role in enzymatic activity formation, which can influence the expression of microorganisms or limit their activity in the rhizosphere^43^.

### Influence of physicochemical parameters on the enzymatic activity of forest soil

Microbial activity is largely dependent on the habitat and tree species, and the decomposition processes occurring in the soil environment are controlled by the quantity and quality of biomass supplied^44,45^. In our study, higher enzymatic activities of β-D-cellobiosidase and arylsulphatase were recorded in soils of deciduous stands. The fractional composition of soil organic matter (SOM) is correlated with enzymatic activity due to the close relationship between microbial decomposition processes of organic matter and the soil enzymes that catalyze these processes^19,46,47^. A strong correlation was noted between the light fraction of soil organic matter (free and occluded) and the activity of selected enzymes. Enzymatic activity in soil is strongly dependent on the availability of substrates, i.e., organic compounds that can be transformed by enzymes. The light fraction promotes rapid growth and activity of microorganisms, which in turn leads to an increased enzyme production^19^. Microorganisms derive energy from readily available organic compounds, which drives enzymatic activity. The heavy fraction is characterized by higher stability and lower energy availability for microorganisms, leading to reduced microbial activity and lower enzyme production^28,48^. In terms of basic chemical properties, fractional composition of SOM and enzymatic activity, soils of beech stands are closer to those of coniferous stands than to those of deciduous stands. Beech litter in stands developing on acidic, carbonate-free soils leads to soil acidification, similar to that of coniferous litter^28,49^. The high content of lignin and other resistant to degradation organic compounds in beech organic debris is conducive to lowering soil pH. Lower soil pH inhibits the activity of many microorganisms responsible for the rapid decomposition of organic matter, which in turn slows down the nutrient cycle, making soil chemical properties similar to those observed in soils influenced by pine stands^50–52^. Carbonate-free soils under beech stands, like those under conifers, tend to accumulate organic compounds that are less susceptible to decomposition and relatively resistant to mineralization^16,53^. This leads to the formation of a higher accumulation of weakly mineral particle-bound SOM fractions, resulting in reduced nutrient availability and slower organic matter cycling, making these soils similar to soils under conifers^52^.

## Conclusions

Soils influenced by deciduous species differ from soils under coniferous stands in terms of the proportion of the various SOM fractions. Coniferous species such as pine and larch, due to their ability to lower soil pH through root secretions and organic precipitation, lead to a slowdown in the processes of decomposition and mineralization of SOM, so that the share of the light fraction of SOM increases. The presence of a higher proportion of the heavy fraction of soil organic matter (SOM) in the soils of beech, oak and hornbeam stands is due to the peculiarities of organic residues, microbial processes and interactions between organic matter and mineral components of soil. The high activity of microorganisms in the soils of deciduous stands, confirmed by enzymatic activity, leads to the efficient decomposition of organic compounds and the formation of stable compounds that constitute the heavy fraction of SOM. Analysis of the fractional composition of SOM in soils under different stands allowed us to assess which tree species most favorably influence soil properties including enzymatic activity, which is of direct relevance to sustainable forest management. The results we obtained can be used in maintaining soil fertility and forest ecosystem health. Studies of the fractional composition of SOM can serve as an indicator of changes in forest ecosystems as a result of climate change, changes in forest management or the effect of human activities.

## Data Availability

The data that support the findings of this study are not openly available due to reasons of sensitivity and are available from the corresponding author upon reasonable request- karolina.staszel@urk.edu.pl.
